# Reversible cerebral vasoconstriction syndrome in a patient taking citalopram and Hydroxycut: a case report

**DOI:** 10.1186/1752-1947-5-548

**Published:** 2011-11-10

**Authors:** Gregory L Cvetanovich, Pankajavalli Ramakrishnan, Joshua P Klein, Vikram R Rao, Allan H Ropper

**Affiliations:** 1 Department of Neurology, Brigham and Women's Hospital, 75 Francis Street, Boston, MA 02115, USA; 2 Department of Neurology, Critical Care Neurology, Massachusetts General Hospital, 55 Fruit Street, Boston, MA 02114, USA

## Abstract

**Introduction:**

Reversible cerebral vasoconstriction syndrome presents with thunderclap headaches accompanied by mild neurologic deficits and is characterized by multifocal narrowing of the cerebral arteries that resolves over days to weeks. This syndrome may be idiopathic or occur in special contexts, most often involving adrenergic or serotonergic overactivity. To the best of our knowledge, reversible cerebral vasoconstriction syndrome has not previously been reported in association with Hydroxycut use in the literature.

**Case Presentation:**

We report the case of a 65-year-old Caucasian woman on longstanding citalopram who developed reversible cerebral vasoconstriction syndrome two weeks after beginning to take the weight-loss supplement Hydroxycut.

**Conclusion:**

There are sparse data about the safety of herbal supplements such as Hydroxycut, even though the Food and Drug Administration has banned some herbal ingredients, such as ephedra, that were in this preparation in the past. This case highlights the importance of considering herbal supplements and potential drug interactions in the genesis of otherwise unexplained reversible cerebral vasoconstriction syndrome.

## Introduction

Reversible cerebral vasoconstriction syndrome (RCVS) is the term for a group of rare syndromes characterized by multifocal narrowing of the cerebral arteries that resolves over the course of days to weeks [1]. Patients present with sudden, severe "thunderclap" headaches that may be accompanied by neurologic deficits [1]. Clinical situations associated with the development of RCVS include pregnancy or the postpartum period and various medications and illicit drugs [2]. RCVS is diagnosed on the basis of this clinical presentation, exclusion of other causes of thunderclap headache such as subarachnoid hemorrhage and cerebral vasculitis by cerebrospinal fluid analysis, documentation of multifocal vasoconstriction of the cerebral arteries by angiography, and of reversibility of the vasoconstriction within 12 weeks of onset, although there may be permanent neurologic injury if stroke occurs secondary to vasospasm [1]. Treatment has included calcium channel blockers [3,4] or magnesium [5], and discontinuation of potential triggers for RCVS, particularly adrenergic or serotonergic compounds.

We report the case of a patient on longstanding citalopram who developed RCVS two weeks after beginning to take the weight-loss supplement Hydroxycut, and we review the literature identifying factors associated with development of RCVS.

## Case Presentation

A 65-year-old Caucasian woman presented to her local hospital with sudden-onset, bifrontal, pounding headache described as "getting hit in the head with an axe." The headache was the worst of her life and did not improve after she took acetaminophen, caffeine, and butalbital. There was hyperacusis, photophobia and nausea. Noncontrast head computed tomography (CT) and brain magnetic resonance imaging (MRI) at the time of admission were normal and she was treated with prednisone for presumed intractable migraine. Aside from a similar but milder headache one week prior to her current presentation, she reported only a sparse past history of migraines that ceased after her hysterectomy and no family history of migraines or strokes. She had hyperlipidemia treated with simvastatin 40 mg daily, lumbar spinal compression fractures, multiple miscarriages and depression that had been treated for several years with citalopram 20 mg daily. On further questioning, our patient reported taking the weight-loss supplement Hydroxycut beginning two weeks prior to her thunderclap headache. On admission, her body mass index was 22.3, and she was normotensive on lisinopril 10 mg daily. She had not previously been on lisinopril, which was presumably initiated at the outside hospital for prednisone-induced hypertension. We held the lisinopril for the duration of her hospitalization given her normal to low blood pressures. Her fasting lipid panel revealed cholesterol 223 mg/dL, triglycerides 141 mg/dL, high density lipoprotein 61 mg/dL, low density lipoprotein 134 mg/dL, very low density lipoprotein 28 mg/dL and lipoprotein(a) 6 mg/dL.

**Figure 1:**
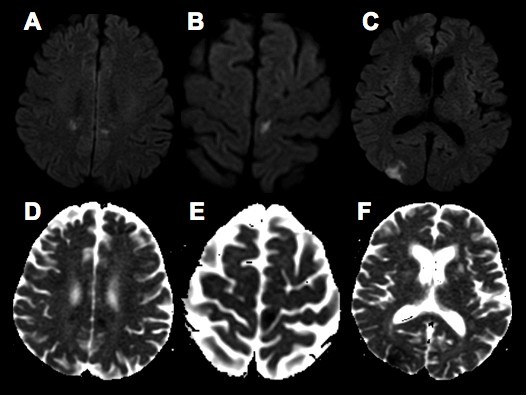
**RVCS-related ischemic strokes**. Diffusion-weighted MRI (A, B, C) and apparent diffusion coefficient maps (D, E, F) revealed lesions in the right occipital lobe and bilateral anterior cerebral artery territories consistent with ischemic strokes.

**Figure 2:**
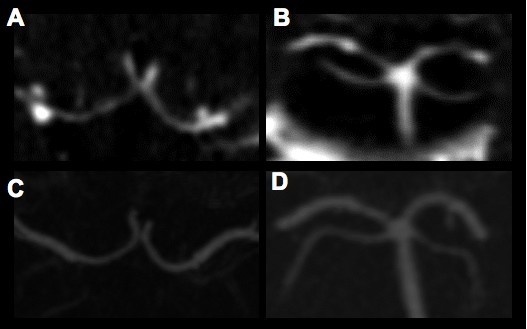
**RCVS on computed tomography angiography**. CTA obtained during hospitalization showed multifocal segmental vasoconstrictions most prominent in the bilateral anterior (A) and posterior (B) cerebral arteries. Follow-up CTA six weeks after discharge revealed marked resolution of cerebral artery vasoconstriction, shown here for anterior (C) and posterior (D) cerebral arteries.

Two days after admission, she developed bilateral leg weakness and left-sided visual disturbances that she described as "blank lines." A repeat MRI revealed areas of restricted diffusion consistent with acute infarcts in the bilateral anterior cerebral artery territories and in her right occipital lobe (Figure 1). The following investigations were unrevealing: hypercoagulability studies, rheumatic and vasculitic screening labs, magnetic resonance venography, transthoracic echocardiogram with bubble contrast, and Holter monitoring. LA lumbar puncture, performed while our patient was being treated with prednisone, revealed 0 white blood cells (WBC), 48 red blood cells (RBC), cerebrospinal fluid (CSF) protein 27 mg/dL, glucose 81 mg/dL and no xanthochromia. CT angiography (CTA) was obtained, which revealed multifocal segmental cerebral artery vasoconstriction, most prominent in the bilateral anterior and posterior cerebral arteries (Figures 2A and 2B).

We made the diagnosis of RCVS and began treatment with nimodipine 30 mg three times daily. Over the subsequent days, her headache resolved and her vision and leg weakness improved. Our patient's blood pressures at admission and prior to starting nimodipine were 92-116/54-58 mmHg on no antihypertensive medications. After beginning nimodipine for RCVS, her systolic blood pressures ranged from the high 80s to low 100s (mmHg). We administered intravenous fluid bolus as needed to keep her systolic blood pressure above 90 mmHg, in an effort to balance maintaining adequate cerebral perfusion while continuing nimodipine treatment for RCVS. Our patient tolerated this well without any clinical decline or symptomatic hypotension.

She was discharged on nimodipine and advised not to take Hydroxycut and citalopram, which had been discontinued when a diagnosis of RCVS was first suspected. At the time of discharge, her systolic blood pressures remained in the 90s to low 100s mmHg. Therefore, she was advised to measure her blood pressure at home and take nimodipine only if systolic blood pressure was over 100 mmHg. Following discharge, our patient experienced no headaches and no recurrence of her presenting symptoms. At a follow-up appointment, she had no residual leg weakness and significant improvement of her left visual field deficit, although she reported that her vision had not returned to her baseline. CTA performed six weeks after discharge showed marked resolution of cerebral vasoconstriction, confirming the diagnosis of RCVS (Figures 2C and 2D).

## Discussion

This case illustrates the cardinal features of RCVS: thunderclap headache, lack of subarachnoid hemorrhage by CSF and radiographic analysis, ostensible exclusion of cerebral vasculitis by CSF and systemic testing, and angiographic demonstration of multifocal segmental cerebral artery vasoconstriction that resolves with time or calcium channel blocker treatment. It also exemplifies ischemic strokes as complications of RCVS [6], emphasizing the delicate balance between maintenance of adequate cerebral perfusion pressure to avoid watershed infarcts while using calcium channel blockers to mitigate against worsening vasoconstriction [2].

The other aspect of RCVS treatment is identification and discontinuation of the potential triggers of RCVS. The clinical settings for RCVS include pregnancy and the postpartum state, serotonergic and sympathomimetic drugs and tumors, direct or neurosurgical trauma, hypertension, primary headache disorders such as migraine and other miscellaneous conditions such as hypercalcemia and porphyria [1,2]. Regardless of etiology, RCVS is thought to occur due to perturbation of cerebral vascular tone [1].

Although amphetamine-related weight-loss supplements and selective serotonin reuptake inhibitors including citalopram have been associated with RCVS [1,7-9], Hydroxycut has not previously been implicated. It is impossible to prove causality, but the temporal relationship between the patient's initiation of Hydroxycut and development of RCVS and the rapid reversal of symptoms and vasospasm following cessation implicate the supplement as a contributing cause in this case. Citalopram may have acted in concert with the newly-initiated Hydroxycut to cause this patient's RCVS, though the fact that she tolerated citalopram well for several years before developing RCVS argues against the antidepressant drug as the sole trigger.

Hydroxycut is among the most popular weight-loss supplements with nine million units sold in 2008 in the US alone [10] and, like most herbal supplements, contains poorly characterized components whose side effect profiles are ill-defined [11]. After Hydroxycut was introduced in 2002, the first adverse events were probably related to its ephedra component, which was banned by the Food and Drug Administration (FDA) in 2004 for causing severe cardiovascular, central nervous system and hepatic toxicity [12]. Since the removal of ephedra from Hydroxycut in 2004, published adverse events have included numerous cases of hepatotoxicity [13], one case of rhabdomyolysis [14] and one case of hypertensive retinopathy [15]. In May 2009, the FDA warned the public about Hydroxycut hepatotoxicity, prompting the manufacturer to withdraw all Hydroxycut products [16].

Recently, a new Hydroxycut formulation has been introduced, and it is this one that our patient with RCVS was taking. Based on the tendency of sympathomimetic agents to trigger RCVS, caffeine may have been the ingredient responsible for RCVS as the usual daily dose of Hydroxycut contains 400 mg of caffeine per day [17], which is equivalent to four cups of coffee. Caffeine is known to cause cerebral vasoconstriction [18], although it has not been associated with RCVS. Other components of the new Hydroxycut formulation may have contributed, although to our knowledge none has vasoconstrictive properties.

## Conclusion

We report a case report of a patient on longstanding citalopram who developed RCVS two weeks after beginning to take the weight-loss supplement Hydroxycut. Given the sparse data about the efficacy and safety of herbal supplements such as Hydroxycut, it is advisable to consider the potential roles of dietary supplements and drug interactions in cases of otherwise unexplained cerebrovascular disease.

## Consent

Written informed consent was obtained from the patient for publication of this case report and any accompanying images. A copy of the written consent is available for review by the Editor-in-Chief of this journal.

## Competing interests

The authors declare that they have no competing interests.

## Authors' contributions

GC drafted the manuscript. PR and JPK assisted with neuroradiology. All authors analyzed and interpreted patient data and read and approved the final manuscript.
